# IRE1α Stands astride Many Paths to Insulin Production

**DOI:** 10.1371/journal.pbio.1002278

**Published:** 2015-10-15

**Authors:** Caitlin Sedwick

**Affiliations:** Freelance Science Writer, San Diego, California, United States of America

## Abstract

A new study shows that components of the unfolded protein pathway are needed to help manage the production of vast amounts of insulin by pancreatic β cells in response to glucose stimulation. Read the Research Article.

Following a meal, blood glucose levels spike. Specialized secretory cells found within pancreatic islets, called β cells, respond to this by secreting the hormone insulin, which stimulates glucose uptake by skeletal muscle and fat tissues. This insulin-stimulated glucose uptake is essential to energetic homeostasis and also serves to protect body cells from toxicity due to high concentrations of extracellular glucose. Thus, conditions that impair β cell insulin production or cause the loss of these cells—as occurs with some forms of diabetes mellitus—can lead to metabolic derangement and even death. For obvious reasons, therefore, the mechanisms regulating β cell insulin production are under intense study. In their paper published this month in *PLOS Biology*, Justin Hassler, Randal Kaufman, and colleagues offer new insights into this process.

Glucose stimulates both insulin granule release and massive new synthesis of insulin to replace the secreted hormone (Fig 1). Replenishment of β cell insulin stores is accomplished through activation of the insulin gene to produce mRNAs encoding preproinsulin, which must then be translated and processed within the endoplasmic reticulum (ER) to generate first proinsulin and then mature insulin protein. Recent studies have shown that the manufacture and processing of these huge amounts of protein actually stresses the cell’s secretory pathway and activates the unfolded protein response (UPR), an adaptive response that helps cells expand and adapt their secretory pathway to prevent the accumulation of unfolded protein within the ER.

**Fig 1:**
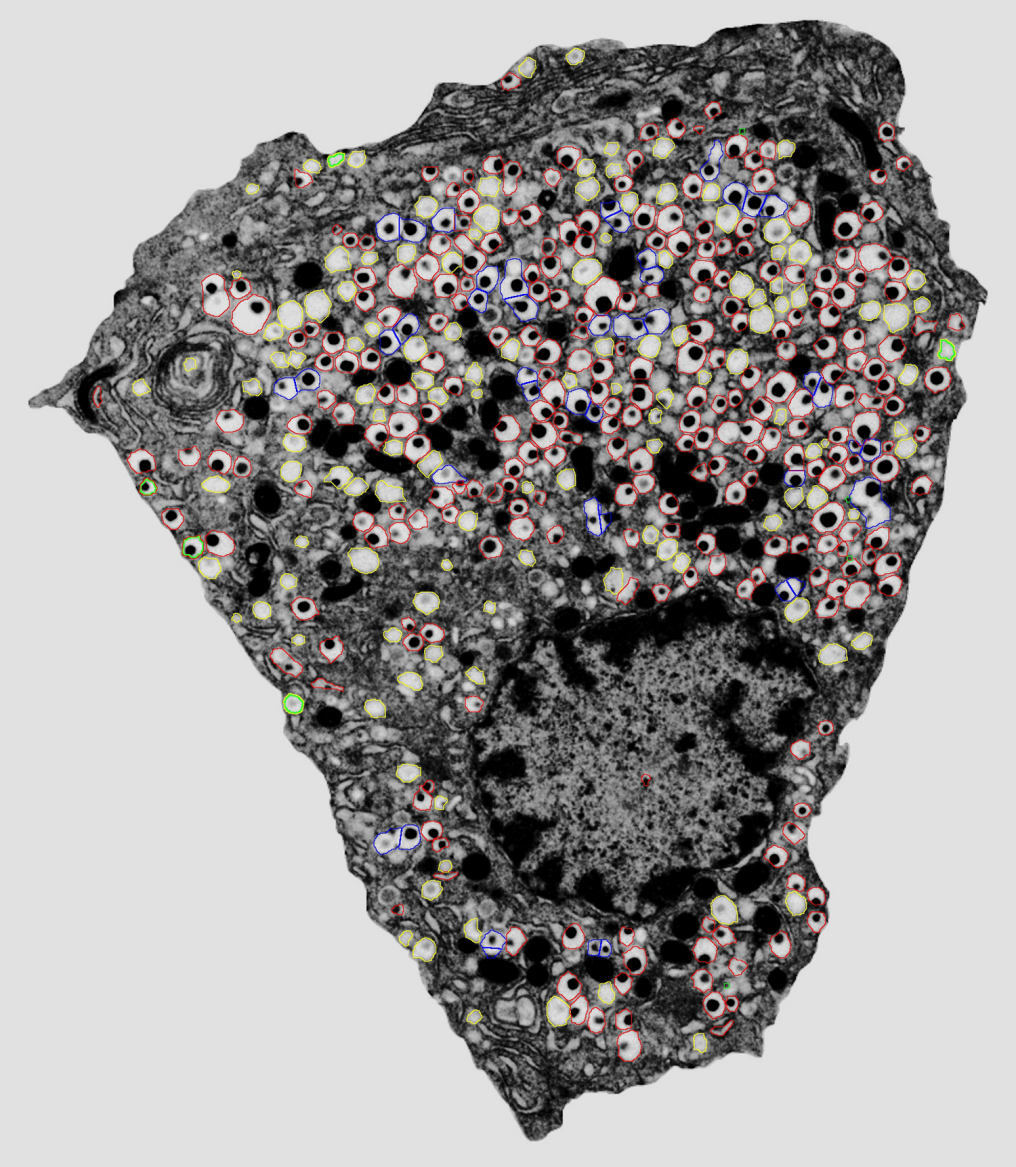
Electron micrograph of a beta cell. Insulin and zinc crystals are stored in the center of the secretory granules. There are approximately 10,000 secretory granules/beta cell. Upon release, the translation of insulin mRNA is stimulated to replenish the granule pool. *Image Credit: NASA, wikimedia.org*

A critical component of the UPR in many cell types is a dual-function protein known as IRE1α, which is autophosphorylated upon initiation of ER stress. Autophosphorylation activates IRE1α’s endonuclease activity, allowing it to cleave messenger RNAs to reduce the folding burden on the ER. Active IRE1α also cleaves the mRNA encoding a transcription factor called XBP1, generating a more potent version that strongly up-regulates expression of many other proteins associated with the UPR. Given its prominent function in the UPR, scientists suspect IRE1α is important for β cell insulin secretion, and studies in embryonic β cells support this hypothesis. However, IRE1α knockout is embryonic lethal in mice, which has hampered efforts to directly test the idea.

To tackle this problem, Hassler et al. used Cre/Lox technology to create transgenic mice in which IRE1α could be deleted specifically in adult animals’ β cells. Mice with β cell-deleted IRE1α showed a diabetic phenotype, with lower basal levels of proinsulin and insulin, less insulin secretion after meals, and therefore elevated blood glucose levels after feeding compared to wild-type mice. This suggested that IRE1α is in fact important for helping β cells manage insulin production, so the authors next investigated the reasons for this defect.

Examination of IRE1α-deleted pancreatic islets showed that the defects in insulin production did not originate at the level of insulin gene transcription. Instead, it occurred because of a block in insulin mRNA translation and impaired processing of insulin precursor proteins in the ER. IRE1α-deleted pancreatic islets showed significant signs of ER stress, indicating that IRE1α deficiency may block insulin production—at least in part—by interfering with β cell ER integrity and function.

For a more in-depth look at the effects of IRE1α deficiency in β cells, the authors examined the full complement of RNAs expressed in the cells using mRNA sequencing (mRNA-Seq). Analysis of these data uncovered hundreds of genes whose expression is coordinately regulated by IRE1α and high levels of glucose, many of which had not previously been identified as part of the IRE1α pathway. For example, compared to wild-type islets, IRE1α-deficient islets showed impaired expression of 141 genes when exposed to high levels of glucose. IRE1α is known to up-regulate expression of several genes indirectly through processing of XBP1, but of the 141 impaired genes, only 22 are known targets of XBP1. Subsequently, functional studies confirmed that XBP1 cleavage by IRE1α is indeed essential to expand ER capacity for insulin processing. However, they also demonstrated that IRE1α is required for diverse other cellular tasks, including processing of preproinsulin to insulin and ribosome recruitment to the ER.

Interestingly, 368 genes were coordinately up-regulated by IRE1α deficiency and high glucose. Among these were several that are known to induce or exacerbate oxidative stress. Consistent with this, IRE1α-deficient islets exhibited many signs of β cell oxidative stress. In addition, the authors found that food laced with antioxidants could ameliorate the diabetic phenotype in mice with β cell-deleted IRE1α. This suggests oxidative stress impairs insulin production by these animals.

Experiments with human islets indicated that, as in mice, IRE1α regulates proinsulin levels without affecting insulin gene expression. Taken together, these data suggest a requirement for IRE1α and XBP1 in many processes that affect glucose-mediated stimulation of insulin production by β cells—a finding that could strongly impact efforts to target this pathway for treatment of diabetes mellitus.

## Abbreviations

ER: endoplasmic reticulum
mRNA-Seq: mRNA sequencing
UPR: unfolded protein response

## References

[1] Hassler JR, Scheuner DL, Wang S, Han J, Kodali VK, Li P, et al The IRE1α/XBP1s Pathway Is Essential for the Glucose Response and Protection of β Cells. PLoS Biol. 2015;13(10): e1002277 doi: 10.1371/journal.pbio.1002277 26469762 10.1371/journal.pbio.1002277 PMC4607427

